# Serum Levels of Cyfra 21 in Patients with Benign and Malignant Salivary Gland Tumors

**Published:** 2017-07

**Authors:** Naghmeh Jeiroodi, Seyed Mohammad- Javad Aslani, Bijan Khademi, Mahyar Malekzadeh, Zohreh Jaafari- Ashkavandi

**Affiliations:** 1 *Department of Oral and Maxillofacial Pathology, School of Dentistry, Shiraz University of Medical Sciences, Shiraz, Iran.*; 2 *Student Research Committee, School of Dentistry, Shiraz University of Medical Sciences, Shiraz, Iran. *; 3 *Otolaryngology Research Center, Department of Otolaryngology, Shiraz University of Medical Sciences, Shiraz, Iran.*; 4 *Institute of Cancer Research, School of Medicine, Shiraz University of Medical Sciences, Shiraz, Iran.*

**Keywords:** Adenoid cystic carcinoma, Cyfra 21, Mucoepidermoid carcinoma, Pleomorphic adenoma, Serum, Salivary gland.

## Abstract

**Introduction:**

Cyfra 21 is a serum-soluble fragment of cytokeratin19. Increased Cyfra 21 serum levels and their benefit as a tumor marker have been shown in some malignancies. This study aimed to evaluate the serum levels of Cyfra 21 in patients with benign and malignant salivary gland tumors.

**Materials and Methods:**

In this cross-sectional study, the serum level of Cyfra 21 in 44 patients with malignant salivary gland tumors and 16 cases of pleomorphic adenoma were compared with 28 healthy controls using enzyme-linked immunosorbent assay (ELISA). Data were analyzed statistically using the Kruskal Wallis test, analysis of variance (ANOVA) and Spearman’s correlation tests.

**Results:**

Mean serum levels of Cyfra 21 were 0.135 ± 0.285 ng/ ml in the control group, 0.167 ± 0.142 ng/ ml in patients with pleomorphic adenoma and 1.059 ± 3.251 ng/ml in patients with malignant salivary gland tumors. There was no significant difference among groups. Cyfra 21 levels did not correlate with location of tumor, clinical stage or cigarette smoking.

**Conclusion:**

Results of the present study showed no significant difference in Cyfra 21 serum level in salivary gland tumors compared with normal individuals. In addition, Cyfra 21 serum level was not sufficiently sensitive to function as a tumor marker in salivary gland tumors.

## Introduction

Salivary gland tumors (SGTs) are important features of head and neck pathology. Pleomorphic adenoma (PA) is the most common SGT, constituting about 70% of neoplasms originating from the salivary glands. After PA, mucoepidermoid carcinoma (MEC) and adenoid cystic carcinoma (ADCC) are the next most common malignant tumors, constituting 35% and 20% of SGTs, respectively. Some malignant SGTs have slow proliferation rates and low aggressive behavior, while others are extremely invasive and metastasize easily (1).

Computed tomography (CT) scan, magnetic resonance imaging (MRI) and fine needle aspiration (FNA) are commonly used for the detection and diagnosis of SGTs and are corroborated by histopathologic confirmation of tumors (2). As some tumors have similar clinical and histopathological features, their diagnosis is difficult. Tumor markers are specific molecules expressed by neoplastic and transforming cells, as well as some normal cells. Use of a suitable tumor marker which is expressed in an appropriate amount into the serum allows earlier diagnosis of particular neoplasms (3). It is important to find a marker with sufficient sensitivity and specificity in a particular neoplasm for early diagnosis (4,5).

Cytokeratins (CK) are considered to be important diagnostic tools for detecting cancer. CKs are expressed in different types of epithelial cell, and increased rates of CKs are seen in malignant epithelial tumors. Enrieder et al. demonstrated increased serum levels of CKs in patients with colorectal cancer (6). In another study, serum levels of CK8 increased with tumor progression in patients with non-small cell lung cancer (NSCLC) (7). Cyfra 21, with a molecular weight of 40 KDa, is the soluble fragment of CK19. Two monoclonal antibodies against CK-19 fragments are found in serum (8,9). Previous studies showed an increased serum level of Cyfra-21 and its sensitivity as a tumor marker in patients with head and neck carcinomas, oral squamous cell carcinoma (OSCC) and esophageal cancer (10-12). This marker has positive relationships with tumor mass and also with tumor necrosis in patients with head and neck cancers. Furthermore, its serum level had a predictive value for detecting head and neck malignancies with a sensitivity of 50% (10).

Overexpression of CK19 has previously been shown in malignant SGTs and hepatocellular carcinoma (10,13). Furthermore, an elevated CK19 expression has been observed in MEC, ADCC and undifferentiated carcinomas. In some studies, increased Cyfra 21 serum expression has been detected in a few cases of SGTs associated with head and neck neoplasms (10). However, adequate sample sizes of SGTs, as an independent group of cancers, have not been investigated. Therefore, considering the importance of finding an appropriate tumor marker for detecting and following up these neoplasms, the present study aimed to evaluate Cyfra 21 serum level in patients with benign and malignant SGTs.

## Materials and Methods

In this retrospective and cross-sectional study, 60 samples of serum from patients with SGTs, including 16 PA, 5 ADCC, 33 MEC, and six acinic cell carcinomas (ACC), as well as 28 serum samples from healthy individuals who referred to the Khalili Hospital, affiliated to Shiraz University Medical Sciences, from 2013 to 2015, were enrolled. 

All diagnoses were confirmed by histopathologic evaluation. The patients had no history of any surgical, chemo- or radio-therapeutic treatments or other cancers. The clinical and pathological features of the patients, including type, site and stage of tumor as well as the patient’s age, gender and smoking habits were recorded. 

The blood samples were centrifuged at 4°C and the serum was kept at −80°C before use. Anti Cyfra 21 antibody (CanAg, Cat no. ABIN 414690, USA) was used with a sandwich enzyme-linked immunosorbent assay (ELISA) method, according to the manufacturers’ instructions.

*Statistical analysis*

A Kruskal–Wallis test was used to compare the serum level of Cyfra 21 in the study groups. Analysis of variance (ANOVA) and Spearman’s Correlation tests were used for assessing the relationship between Cyfra 21 level and patient variables. P-values ≤ 0.05 were considered statistically significant.

## Results

In this study, 44 cases of malignant tumor, 16 cases of PA and 28 normal cases were investigated. The patients included 32 males and 56 females, ranging from 46 to 54 years of age, with a mean age of 49.67 years. Of the 60 patients, 10 (16.66%) had a history of smoking. Baseline data are illustrated in Tables 1 and 2. Mean serum levels of Cyfra 21 were 0.135 ± 0.285 ng/ml in the control group, 0.167 ± 0.142 in PA and 1.059 ± 3.251 ng/ml in patients with malignant SGTs (Table.1). The Kruskal–Wallis test showed no significant difference in Cyfra 21 levels between patients and healthy individuals (P=0.796). Also, there was no significant correlation between Cyfra 21 concentration and clinical stage, smoking, tumor size, tumor location or patient age, using Spearman’s correlation (P>0.05 in all cases).

**Table 1:** Baseline data and Cyfra 21 serum levels in benign and malignant salivary gland tumors and control group

**Group (N)**	**Gender** **(M:F)**	**Age** **(Mean ± SD)**	**Cyfra 21** **(Mean ± SD)**	**Min**–**Max**
Control (28)	11:17	49.79 ± 17	0.135 ± 0.285	0–1.415
PA (16)	7:9	46/27 ± 15	0.167 ± 0.142	0–0.400
Mal. Tumors (44)	14:30	52/95 ± 17	1.059 ± 3.251	0–19.44
MEC	9:24	53.47 ± 16	0.698 ± 1.79	0–9.69
ADCC	3:2	50.61 ± 15	4.44 ± 8.45	0–19.44
ACC	2:4	54.68 ± 16	0.225 ± 251	0–0.553

Mal: Malignant, PA: Pleomorphic adenoma, MEC: Mucoepidermoid carcinoma, ADCC: Adenoid cystic carcinoma, ACC: Acinic cell carcinoma

**Table 2:** Location and clinical stage of salivary gland tumors

	**N (%)**	**Malignant**	**PA**
**Location** *			
Major Salivary gland Minor Salivary gland	52 (91.3) 5 (8.8)	36 (87.8%) 5 (12.2%)	16 (100%) 0
**Tumor size**			
T1 T2 T3 T4	4 (9.1) 28 (63.6) 10 (22.7) 2 (4.5)		
**Lymph node involvement**			
NO N1 N2 N3	28 (63.6) 7 (15.9) 9 (20.5) 0 (0)		
**Metastasis**			
Mo M1	41 (93.2) 3 (6.8)		
**Stage**			
1 2 3 4	11 (25) 8 (18.2) 6 (11.36) 20 (45.44)		

* The tumor location was not available for three malignant neoplasms

## Discussion

Cyfra 21 is a soluble fragment of Cytokeratin 19 (a cytoskeletal component of the eukaryotic cells) which has been shown to be overexpressed in tumors of epithelial origin (14). Some studies have demonstrated increased levels of this protein in the sera of patients with SGTs associated with cancers of the head and neck (10). In the present study, SGTs were studied as an independent group. The results of this study show an increase in the Cyfra 21 level in malignant tumors in comparison with healthy individuals and also with benign SGTs. However, the difference was not statistically significant. Immunohistochemical analyses of CK19 in SGTs revealed the overexpression of this protein in the luminal and intermediate cells of MEC (15). Furthermore, Altemini et al. demonstrated that CK19 was expressed in atypical luminal cells of ex-PA (16). The protein was also shown to be overexpressed in epidermoid cells in MEC, and also in luminal cells in acinic cell carcinoma in previous studies (17,18).

Many researchers have demonstrated that during malignant transformation, cell death and necrosis might lead to the release of CK fragments from malignant epithelial tumors to the circulation, consequently increasing the Cyfra 21 serum level (19). However, the results of the present study showed that the increased level of Cyfra 21 was not high enough to provide a sensitive and specific tumor marker in our samples. Kosaka et al reported decreased levels of Cyfra 21 in sera and increased CK-19 in tumoral tissues of patients with NSCLC (20). However, levels of Cyfra 21 were shown to increase in lung (21), esophagus and breast cancers by other researchers (22). Doweck et al showed a correlation between the serum level of Cyfra 21 with tumor mass and, to a greater extent, with tumor necrosis in patients with head and neck carcinomas. They also found a closer relationship between the serum levels of Cyfra 21 and tumor mass than that between serum levels of Cyfra 21 and tumor stage. They concluded that the detection of Cyfra 21 may perhaps function as a prior prognostic factor in head and neck cancer (10). In addition, Cyfra 21 overexpression was reported in HNCC and nasopharynx cancer in two independent studies in 1995 and 1996, respectively (23,24). The mean serum level of Cyfra in patients with SCC was ≥5.14 ng/ml compared with >3.3 ng/ml in those with lung cancer and 1.50 ng/ml in those with benign tumor (11, 25). Furthermore, it was shown that serum levels of Cyfra 21 higher than 3.0 ng/ml as the cut-off point could be correlated with poor survival in patients with epithelial ovarian cancer (26). However, in the present study, patients with SGT had a lower mean Cyfra 21 level, which is probably due to the grade of the tumors. There were a number of cases in our study with ACC and low-grade MEC, which did not show significant necrosis, and therefore Cyfra 21 did not increase. However, the few cases of ADCC with a high-grade SGT showed a higher level of Cyfra 21. Although an increased serum level of Cyfra-21 was found in 44 cases of malignant SGTs, non-homogenous types of tumor might have led to non-significant results. Since ADCC, an invasive and aggressive tumor, had a higher mean Cyfra 21 level in comparison with MEC and ACC, evaluating Cyfra 21 in a larger sample size of ADCC might have led to different results. In this study, in line with the findings of Doweck et al, no correlation was found between the serum levels of Cyfra 21 and the stage of disease (10). However, one study has reported that Cyfra 21 had sufficient sensitivity and specificity in the early stages of lung cancer (25). Furthermore, the elevated Cyfra 21 level was considered as a reliable tumor marker regarding its sensitivity in breast cancers (22). Some researchers stated that many tumor markers were not suitable as diagnostic tools because there were several cases of benign tumors that exhibited an abnormal increase in a specific tumor marker (27,28). Kyoko et al reported a 68% and 69% increase in the specificity and sensitivity of Cyfra, respectively, when accompanied by CEA as tumor markers in lung cancer patients, in comparison with 43% specificity and 89% sensitivity if Cyfra 21 was considered alone (21).

## Conclusion

According to the results of the present study in SGTs, the serum level of Cyfra 21 in benign and malignant SGTs was not significantly different from that found in normal individuals. Further studies focusing on high-grade tumors may lead to more precise results.
